# In-Flight Self-Calibration of the STIX Grid Transmission

**DOI:** 10.1007/s11207-026-02739-6

**Published:** 2026-09-10

**Authors:** Paolo Massa, Anna Volpara, Muriel Zoë Stiefel, Erhan Bilgili, Maik Degen, Olivier Limousin, Gordon J. Hurford, Säm Krucker

**Affiliations:** 1 University of Applied Sciences and Arts Northwestern Switzerland, Bahnhofstrasse 6, 5210 Windisch, Switzerland; 2 MIDA, Dipartimento di Matematica, Università di Genova, via Dodecaneso 35, I-16146 Genova, Italy; 3 ETH Zürich, Rämistrasse 101, 8092 Zürich, Switzerland; 4 Université Paris-Saclay, Université Paris Cité, CEA, CNRS, AIM, 91191 Gif-sur-Yvette, France; 5 Hurford Modulated Solution, 442 Larkin Drive, 94510 Benicia, CA USA

**Keywords:** Instrumentation and data management, Flares, spectrum, Spectrum, X-ray

## Abstract

The *Spectrometer/Telescope for Imaging X-rays* (STIX) on board *Solar Orbiter* provides imaging information on the X-ray sources in solar flares by modulating the emitted radiation with pairs of grids made of stacked tungsten layers. Optical measurements of the grids were taken on the ground and utilized to derive an initial model for the grid transmission. In this work, we describe an in-flight self-calibration procedure of the STIX grid transmission below 20 keV. This technique utilizes fully-illuminated pixels of the STIX Coarse Flare Locator (CFL) as a total flux monitor. The results show that the newly established transmission is lower than what was derived from the optical measurements. This is due to imperfections of the tungsten layers which reduce the effective slit width. Pre-flight optical measurements only measure the outer layers leaving these imperfections undetected. Using the new calibration, the photon spectra derived from individual sub-collimators are consistent within ∼ 2% between 8 and 20 keV. While fitted flare temperatures do not change with the new calibration, emission measure values increase by ∼ 13% compared to those obtained with the initial grid measurements.

## Introduction

The Spectrometer/Telescope for Imaging X-rays (STIX; Krucker et al. 2020) is an indirect hard X-ray (HXR) imaging spectrometer onboard ESA’s Solar Orbiter mission (Müller et al. 2020). STIX observes the X-ray bremsstrahlung emission produced by the hottest flare loops and by accelerated electrons (e.g., Tandberg-Hanssen and Emslie 1988; Benz 2017). The spectroscopy capability of STIX is provided by pixelated semiconductor (CdTe) detectors operating in the energy range 4 – 150 keV with an energy resolution of 1 keV (Meuris et al. 2012). Imaging information is encoded by spatially modulating the incident X-rays by means of 30 pairs of tungsten grids placed in front of the detectors. Each grid pair projects a shadow in the shape of a specific Moiré pattern on the corresponding STIX detector (Prince et al. 1988; Hurford 2013). By measuring the contrast (amplitude) and location (phase) of the Moiré pattern, each *sub-collimator* (i.e. a detector-grid pair unit) provides a Fourier component of the X-ray source(s), or in radio astronomy terms, a *visibility* (Massa et al. 2023).

The STIX grids are made of layers of tungsten foils with thicknesses of 33 or 50 μm that are stacked to achieve the required grid thickness of $d=400~\mu\mathrm{m}$ for the 24 sub-collimators with coarsest angular resolution. The thickness of the remaining six sets of grids is either $d=200~\mu\mathrm{m}$ or $d=133~\mu\mathrm{m}$. The grid pattern is etched into each layer. To measure an extended range of source sizes and orientations, the 30 STIX grid pairs are divided into 10 groups of 3 pairs. The groups take 10 different pitch values logarithmically spaced from 38 to 958 μm. Grids with the same pitch have three different orientations separated by 60 degrees (e.g. Krucker et al. 2020). In this way STIX samples 30 specific angular frequencies of the Fourier transform of the flaring X-ray sources. For more detailed information on the STIX imaging system or indirect X-ray imaging systems in general, we refer to Massa et al. (2023) and Piana et al. (2022), respectively.

An extensive calibration of the grid pitch, slit width, and slit orientation, is needed for appropriately taking into account the transmission of the sub-collimator grids for STIX imaging and spectroscopy (see Figure 2 in Massa et al. 2023). The STIX grids have been measured optically and with X-rays before the launch of Solar Orbiter (Kuhar 2018). The obtained values of the grid parameters have been used within the data analysis software up to now (Dickson et al. 2025). The shortcoming of this approach is that the optical measurements provide information only on the top and bottom layers, but not on the accuracy of the stacking of individual layers. Hence, the optically measured slat width is systematically underestimated. Furthermore, in contrast with what was done for the Hard X-ray Telescope (HXT; Kosugi et al. 1991; Sakao 1994) on board the Yohkoh mission (Ogawara et al. 1991), for the Reuven Ramaty High Energy Solar Spectroscopic Imager (RHESSI; Lin et al. 2002), and for the Hard X-ray Imager (HXI; Zhang et al. 2019; Jiang et al. 2023; Su et al. 2024) on board the ASO-S mission (Gan et al. 2019, 2023), the X-ray measurements were not done at small angles relevant for solar flare observations (i.e. within a degree) due to time and financial constraints.

In the current version of the grid transmission correction, which is used in spectroscopic analyses, we assume that the grid geometry is perfect, i.e. that all layers within the same grid have the same slit width and that there are no inaccuracies in the etching and stacking of the grid layers. The assumption of perfect grids is used for a mathematical description of the *internal shadowing* of the grid, which is the reduction of the effective slit width that occurs once X-ray photons are stopped by the vertical side of the grid slats. However, we know that etching produces a slightly different slit width for the different layers (*etching imperfections*) and the stacking process has uncertainties (*stacking imperfections*). Etching and stacking imperfections are expected to be of the order of a few microns. When, due to etching and stacking imperfections, individual layers extend slightly into the slit, the grid transmission is reduced. While the impact of etching and stacking imperfections on the transmission is small for coarse grids, for fine grids (with slit widths of a few tens of microns) it becomes significant. Further, the internal shadowing effect can be reduced by etching and stacking imperfections of the grids’ layers. Etching and stacking imperfections are random inaccuracies of the grid manufacturing. In addition, systematic imperfections causing, e.g., tilted slat profiles, could be present.

To provide an accurate calibration of the effective transmission of the STIX sub-collimators, which also includes effects resulting from etching and stacking imperfections, we use solar flare data provided by STIX. Specifically, following the approach by Stiefel et al. (2025), we consider measurements of the X-ray flux through the grid pairs in combination with the total flux measurements provided by fully-illuminated pixels from the STIX Coarse Flare Locator (CFL; Krucker et al. 2020). The effective grid transmission for each sub-collimator is derived as the ratio of the transmitted flux over the total flux measurement provided by the CFL. With the self-calibration approach discussed in this work we measure the combined transmission of both front and rear grid for each sub-collimator, which is the relevant parameter for spectroscopic analyses. For imaging, this self-calibration will provide an effective slit-to-pitch ratio averaged over the two grids of each sub-collimator, which can be used to calibrate the visibility amplitudes (Massa et al. 2023).

We perform simulation tests to demonstrate that stacking and etching imperfections can be responsible for the lower value of transmission that is derived from experimental data relative to that computed by using the on-ground grid characterization and assuming perfect grids. Further, we show with simulations that stacking and etching imperfections can decrease the effect of internal shadowing, in line with what was derived from experimental data. Finally, we show that the new transmission calibration improves the consistency among the data provided by the different sub-collimators and we quantify the impact of the new calibration on spectral analyses of solar flares.

The proposed STIX grid transmission calibration is derived from data recorded in the energy range 10 – 15 keV and is accurate only at low energies (≲ 20 keV). This calibration is not valid at high energies as the effective slit width increases due to transparency of the tungsten grid imperfections. The high-energy grid calibration must be addressed differently from what was done in the present paper as the CFL data statistics at high energies is very low for the considered dataset of flaring events. We will describe the high-energy grid calibration in a follow-up paper. Moreover, the derived transmission calibration is not accurate in the low energy end (below 8 keV) as other systematic effects come into play. These effects are probably due to inhomogeneities in the layers covering the entrance windows, and will be investigated in a future study.

The paper is structured in the following way. Section 2 describes the calibration procedure based on STIX CFL data and shows the resulting transmission calibration for each sub-collimator. Section 3 presents the simulation tests we implemented to qualitatively explain the results derived from experimental data, while Section 4 contains the consistency tests we performed to demonstrate the improvements from the new calibration. Finally, conclusions are drawn in Section 5.

## Self-Calibration Using Flare Data

**Figure 1:**
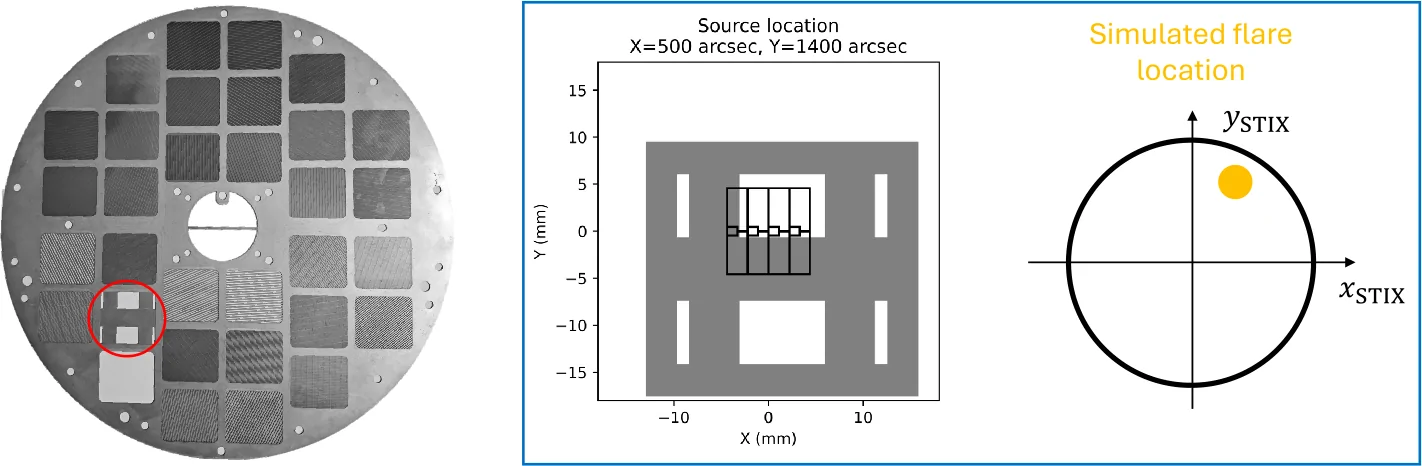
*Left panel:* picture of the front STIX grid, with the CFL window highlighted. *Right panels:* simulation of the CFL shadow that is projected on the detector (*middle*) by a point-like X-ray source located in $\left(x,y)=(500^{\prime \prime },1400^{\prime \prime }\right)$ (*right*). In this case, 3 large CFL pixels are fully illuminated. The simulation has been performed by means of the *pystixsim* library (https://github.com/drhlxiao/pystixsim.git).

In this Section, we present the method we utilized to determine the effective transmission of the sub-collimators using flare observations. Since STIX does not have a total flux monitor, we consider flaring events for which at least one of the CFL detector pixels is fully illuminated. The CFL sub-collimator consists of an “H-shaped” front grid (see the left panel of Figure 1) and of an empty rear grid. For specific locations of the flaring events, the shadow cast by the X-ray radiation over the detector surface covers only a subset of the pixels (right panel of Figure 1). Therefore, a few pixels (up to three) are fully illuminated by the X-ray radiation, and they provide a measurement of the incident total flux. The effective sub-collimators’ transmission can be determined by computing the ratio between the total transmitted flux recorded by the imaging detectors and the total flux measurement provided by the CFL.

### Dataset

At the time of writing, the STIX science flare list^1^ consists of approximately 25’000 flares recorded by STIX between January 1, 2021, and February 28, 2025, with available pixel data (i.e. STIX level 1 science data), and more than 1000 counts in the 4 – 10 keV quicklook profile at peak time. From this dataset, we select events with at least one large fully illuminated CFL pixel, identified by means of a routine implemented in the Interactive Data Language (IDL) software. This routine relies on a lookup table containing the amount of area of the CFL pixels that would be illuminated by a (point-like) X-ray source located at each point of an equispaced grid on the solar disk. Given a specific flare location, the routine determines which pixels are fully illuminated by finding the closest point on the grid and by examining the amount of area of the CFL pixels that would be illuminated from that point.

The location of each flare in the dataset is estimated by means of an IDL procedure that creates a full-disk back-projection map from the visibilities, and then employs VIS_FWDFIT (Volpara et al. 2022) to fit a single elliptical gaussian source and improve the flare location accuracy. The quality of each flare location is assessed by examining the back-projection maps: if any other maximum in the map exceeds 80% of the flare location’s maximum, the quality check fails, and the flare is excluded from this analysis. Note that, for the purposes of this calibration task, it is necessary to consider the flare locations as expressed with respect to the STIX imaging frame reference system (see Section 3 in Massa et al. 2023). This is different from the usual Helioprojective-Cartesian frame (HPC; Thompson 2006) as the telescope is mounted on the side of the spacecraft and observes the Sun as 90^∘^ rotated. Further, the instrument pointing is usually different from solar center and an additional roll angle can be present.

For each event in the dataset, we consider data recorded in the 10 – 15 keV energy range based on the following considerations. The front part of the STIX instrument consists of two beryllium windows shielding the imager from heating and from the low-energy X-ray radiation. The front window is covered with Solar Black, which is the external component of the spacecraft heatshield. This material presents potential inhomogeneities, which would make the transmission different for the different detectors. In that case, it would not be possible to compare the flux recorded by the imaging detectors with that recorded by the CFL, as the basic assumption is that the spectral response of the different sub-collimators is the same (even if not diagonal). Since the Solar Black transmission in the 10 – 15 keV energy interval ranges between ∼ 91% and ∼ 97%, potential inhomogeneities would cause only second order discrepancies in the transmission. The same is not true for lower energies (e.g., 5 – 10 keV), where the Solar Black transmission has a large impact, ranging between ∼ 55% and ∼ 91%. We speculate that the Solar Black is the layer with largest inhomogeneities and we assume that the transmission of all the other layers of the instruments is well characterized. Finally, considering high energy data (≳ 20 keV) for our analysis would lead to a significant drop of the data statistics, thus increasing the uncertainty on the results. This is also due to the fact that the largest flares in the STIX archive cannot be used for this study as they occurred at locations for which none of the CFL pixels is fully illuminated.

For each event in the flare list with accurate associated location, we select a time range around the peak consisting of all time bins for which the total flux value is larger than half of the peak value. In the case where this choice results in a very large time range, we arbitrarily set the maximum duration of the time interval equal to 6 minutes to only cover the main peak of each event. Finally, we discard events with a total number of counts recorded by the 24 coarse-resolution sub-collimators lower than $2 \cdot 10^{5}$ to ensure statistical robustness. Neglecting differences in the pixel counts due to the modulation of Moiré patterns, this threshold ensures an average statistical error of 3% or lower for each individual pixel measurement.

**Figure 2:**
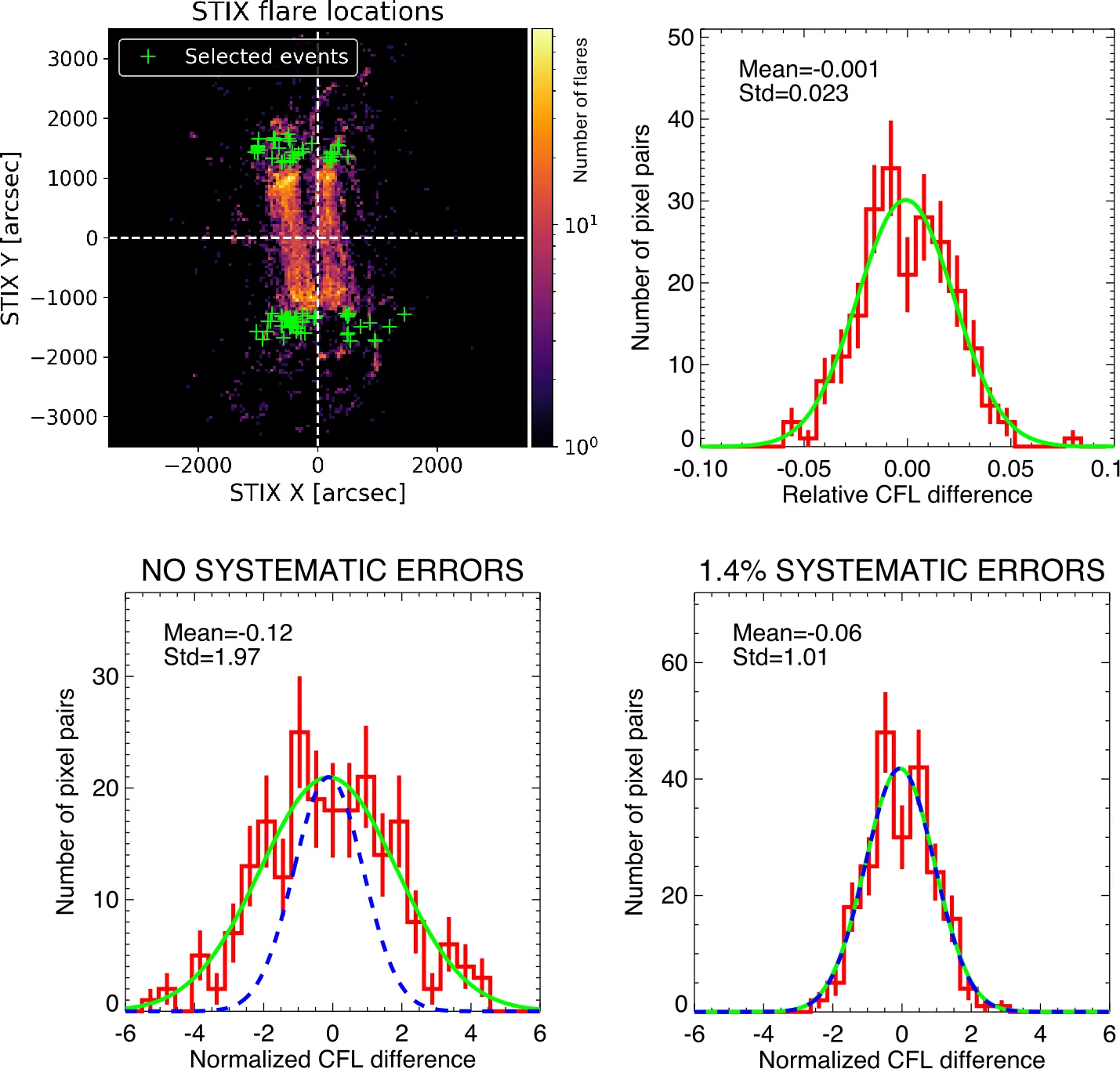
*Top-left panel:* locations of the 91 events with fully illuminated CFL pixels used to self-calibrate the transmission of the STIX sub-collimators (*green crosses*). For reference, a 2D histogram of the locations of ∼ 21 K flaring events observed by STIX between January 1, 2022 and August 30, 2024, is plotted in the background with a logarithmic color map. The locations are expressed with respect to the STIX imaging frame (see main text). *Top-right panel:* histogram of the relative difference of the fully illuminated CFL pixel measurements for the events in our dataset. *Bottom panels:* histograms of the normalized difference of the fully illuminated CFL pixel measurements with (*left*) no systematic errors considered in addition to statistical ones and (*right*) 1.4% systematic errors included. In the histogram panels, *solid green lines* indicate Gaussian fits to the histograms (the fitted mean and standard deviation are reported in the legend), while *dashed blue* lines represent standard normal distributions. Error bars are computed assuming Poisson fluctuations in the bins.

Following this procedure, we select 91 events with at least one fully illuminated large CFL pixel: 1 event has only one fully illuminated large CFL pixel, 27 cases have 2, and 63 show 3 fully illuminated pixels. The selected events are entirely located within the full-sensitivity field of view (FOV) of the instrument (see Section 5 in Massa et al. 2023), so that in the absence of grids, all detector pixels of the imaging sub-collimators would be fully illuminated. The top-left panel of Figure 2 shows the positions of the selected flares, which are plotted in the STIX imaging frame. The events selected for the self-calibration are located in two regions corresponding to absolute values of the Y coordinate around 1500 arcsec. For flares in these regions at least one CFL pixel is fully illuminated. For reference, we plot a 2D histogram of the positions of ∼ 21 K flares taken from the STIX science flare list. This histogram shows two vertical bands corresponding to typical latitudes at which solar flares usually occur. Unfortunately, only a small fraction of the total number of STIX flares can be used for this self-calibration task.

For events with at least two adjacent fully illuminated CFL pixels, we assess the consistency of the recorded count fluxes as follows. For each event, we compute the relative difference of the CFL pixel counts, i.e. the difference normalized by the average value. The corresponding histogram is plotted in the top-right panel of Figure 2. A Gaussian fit to the histogram shows a standard deviation of ∼ 2%. The width of the histogram is caused by both statistical and systematic errors affecting the CFL measurements.

To quantify the amount of systematic errors affecting the data in addition to statistical ones, we compute the normalized difference of the CFL pixel measurements, where the normalizing factor is the sum in quadrature of statistical errors^2^ on the recorded counts. In the case where no systematic errors were present in the data, the corresponding histogram would follow a Gaussian distribution with average and standard deviation equal to 0 and 1, respectively. However, the histogram is wider than a standard normal distribution, its standard deviation being close to 2 (see the bottom-left panel of Figure 2). In the bottom-right panel of the same figure, we compute again the histogram of normalized difference of the CFL pixel measurements, this time adding 1.4% systematic errors in quadrature to the statistical errors of the CFL pixel counts. The percentage value of the added systematic errors is comparable to the statistical error in a single CFL pixel, which varies between ∼ 0.5% and ∼ 1.5%. As expected, the histogram becomes narrower and it nicely matches a standard normal distribution, as highlighted by a gaussian fit. One contributing factor to these systematic errors is the energy calibration based on the Energy LookUp Table (ELUT), which will be briefly described at the beginning of Section 2.2. Based on this self-consistency test, we estimate that the total flux measurements provided by the CFL are accurate up to 2.3%, with even contribution from counting statistics and systematic errors.

### Coarse-Resolution Sub-Collimators

Before deriving the effective transmission for each STIX sub-collimator, we apply the following corrections to the CFL data and to the data recorded by the imaging detectors. First, we subtract background counts produced by the radioactive calibration sources within the STIX instrument by considering appropriate background measurements, and uncertainty on these measurements is propagated. The selected events contain less than 1% of background counts. Therefore, background subtraction does not represent a significant source of uncertainty for this analysis. Second, we normalize the recorded counts by the corresponding detector live time value, and we propagate the live time uncertainty. The live time fraction is on average 0.93, with a standard deviation of 0.07. Hence, even with a conservative estimate of the live time accuracy of several percent, potential errors affecting the live time correction are well below one percent. Finally, we apply an energy calibration based on the ELUT. The STIX native channels (with a width of 0.1 keV) are binned on-board in 32 science channels with a nominal width ranging from 1 to 30 keV. The ELUT contains information about which fine-resolution energy channels are binned onboard to create the (large) science channels. The STIX pixels bin the fine-resolution energy channels in different ways due to the different energy calibration, and the edges of the resulting science channels are slightly different from the nominal ones. Therefore, corrections need to be applied to account for discrepancies across pixels. The applied ELUT correction is 2.8% on average, with a standard deviation of 2.1%. Although we do not have an estimate for the uncertainty of this correction, preliminary assessments suggest that it is of the order of a percent. In summary, out of these three corrections, the ELUT correction is the one introducing the largest uncertainty.

The STIX detectors are partitioned into 12 pixels, 8 of which have a larger area compared the remaining 4 ones (Meuris et al. 2012; Krucker et al. 2020). The large pixels are symmetrically distributed in two rows (*top* and *bottom* row), whereas the small pixels (which will not be considered in this work) are located in the central part of the detector. Denoted by A, B, C, and D the number of counts recorded by the four large pixels of either the top or the bottom row, the total transmitted flux measured by a detector (in counts $\mathrm{s}^{-1}\text{ cm}^{-2}$) is given by (Krucker et al. 2020; Massa et al. 2023)

1 $$ F = \frac{A+B+C+D}{\tau \cdot \mathcal{A}} ~, $$

where τ is the detector live time and $\mathcal{A}$ is the incident area. In the following, we will compute 1 by summing over the top and the bottom row of pixels and by normalizing by the respective area. The transmission of the coarse-resolution sub-collimators is given by

2 $$ t_{\mathrm{coarse}} = \frac{F}{F_{\mathrm{CFL}}} ~, $$

where $F_{\mathrm{CFL}}$ denotes the total flux estimated by the fully illuminated CFL pixels.

**Figure 3:**
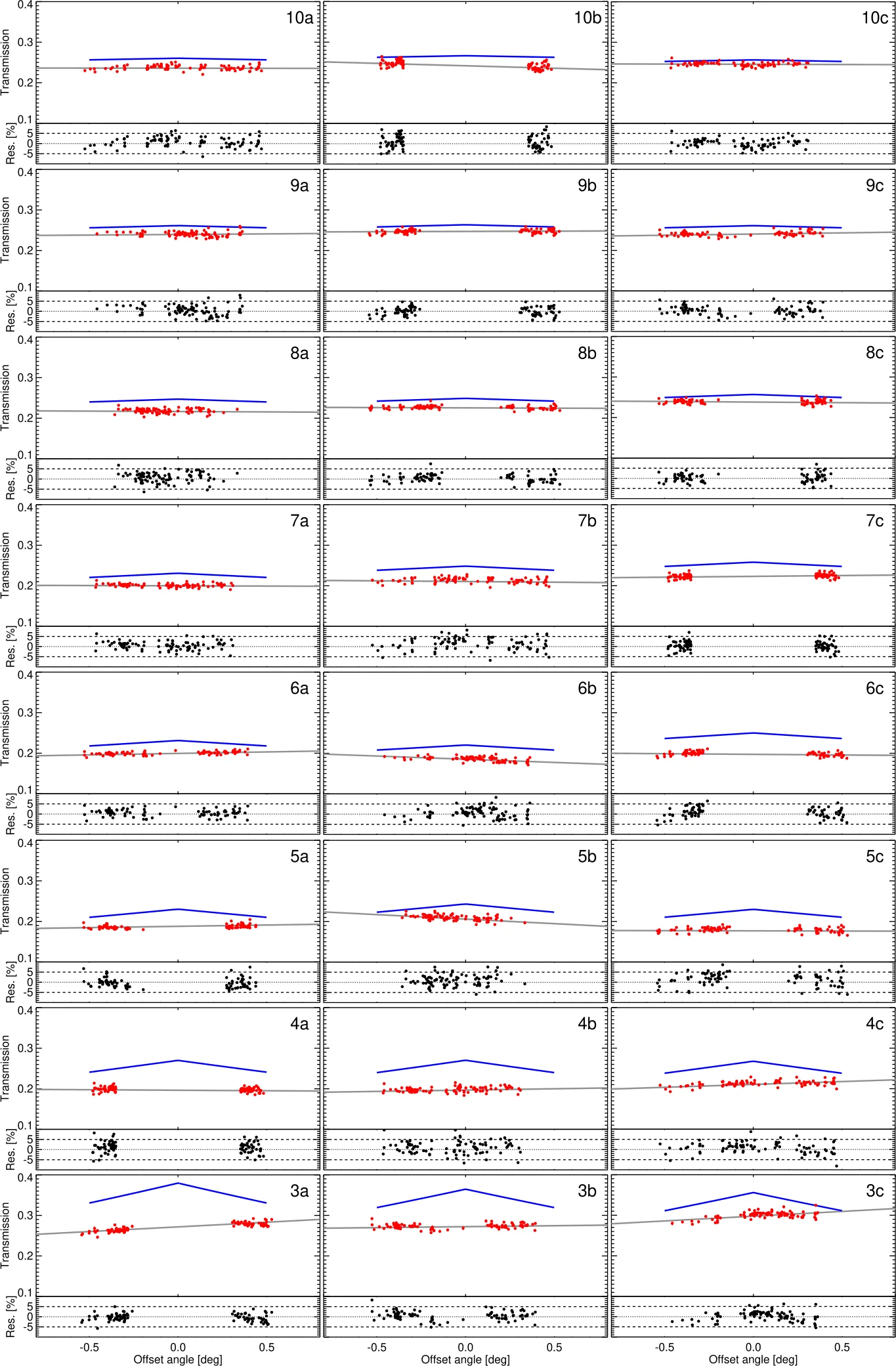
Sub-collimator transmission values estimated using the flaring events in our dataset (*red dots*) plotted as a function of the corresponding offset angle. The *gray lines* represent linear fits to the transmission values derived from solar flare observations, and the corresponding residuals are plotted as *black dots*. The blue lines represent the transmission of ideal sub-collimators without manufacturing imperfections. For calculating the transmission of ideal grids, we considered parameter values derived from the optical grid characterization, and we assumed full grid thickness for the computation of internal shadowing. Each panel refers to a different sub-collimator, whose label is reported in the top-right corner.

**Figure 4:**
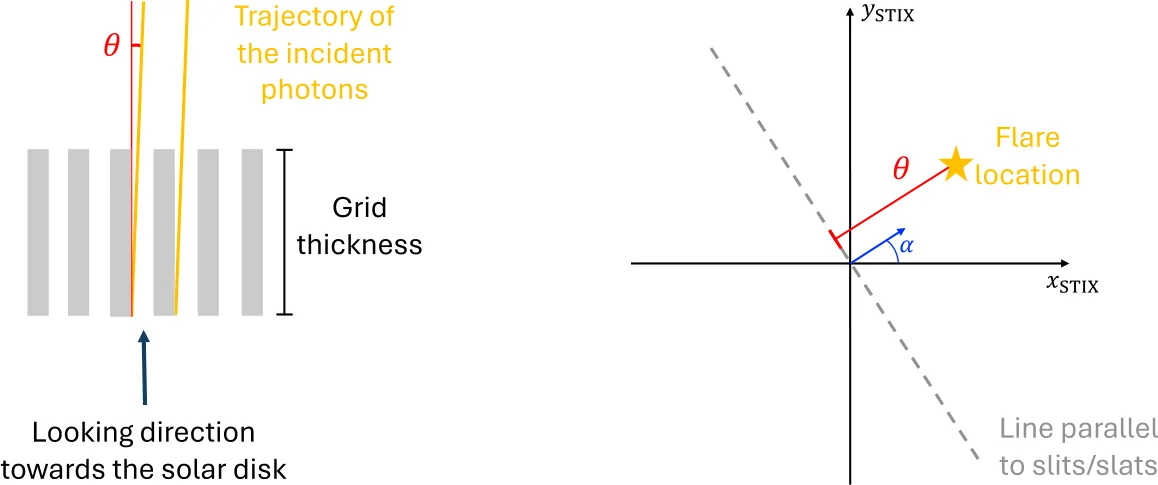
*Left panel:* grid profile in the 2D plane perpendicular to the grid slits/slats. The trajectory of the photons incident on the grid is displayed with *gold lines*, and the corresponding off-axis angle θ is indicated in *red*. *Right panel:* schematic showing how the off-axis angle θ is computed. The flare location is indicated with a *gold star* and the grid orientation angle α is reported in *blue*. The *dashed gray line* represents the line parallel to the slits/slats passing through the origin of the STIX imaging reference frame, while the *red line* indicates the magnitude of θ.

Figure 3 provides an estimate of the transmission of each of the 24 STIX sub-collimators with coarsest angular resolution. These sub-collimators are denoted by a label, which consists of a number ranging from 3 to 10 (the largest the number, the coarsest the sub-collimator resolution), and by a letter (a, b, or c) indicating the orientation of the grid slits. Each panel of Figure 3 refers to a specific sub-collimator and each red dot represents the observed sub-collimator transmission for a single flaring event in our dataset. The transmission values are plotted as a function of the offset angle, i.e. the angle subtended by the flare location perpendicular to the slits/slats. Mathematically, the offset angle is defined as

3 $$ \theta = x_{\mathrm{STIX}} \cdot \cos \alpha + y_{\mathrm{STIX}} \cdot \sin \alpha ~, $$

where $(x_{\mathrm{STIX}},y_{\mathrm{STIX}})$ are the coordinates of the flare location (in arcsec) conceived in the STIX reference frame and α is the orientation angle of the grids (averaged between front and rear) as defined in Massa et al. (2023). As shown in the right panel of Figure 4, the angle α is subtended by the positive x-semiaxis and the direction perpendicular to the grid slits/slats. Consequently, the off-axis angle θ defines the incident angle of the photons in the 2D plane perpendicular to the grid slits/slats (see the left panel of Figure 4). We will discuss below that θ is the relevant angle for the calculation of the internal shadowing of the grid. Note that for sub-collimators whose grids have orientation angle close to 90^∘^ (e.g., 10b, 7c, and 4a), the offset angle becomes $\theta \simeq y_{\mathrm{STIX}}$. Therefore, for these sub-collimators, the offset angles corresponding to the set of 91 events selected in Figure 2 take only two possible values, $\theta \simeq -1500'' \simeq -0.4^{\circ}$ or $\theta \simeq 1500'' \simeq 0.4^{\circ}$. This is clearly visible in Figure 3 for sub-collimators 10b, 7c, and 4a.

In each panel of Figure 3, the gray line represents a linear fit to the observed transmission values. The error bars associated with the observed transmission values reflect only statistical uncertainty, and are very small compared to the observed scatter, which is very likely produced by unknown systematic errors. Therefore, to provide an estimate of the goodness of the linear fit, we express the residuals as the relative difference between the observed and the fitted transmission, normalized by the fitted linear model, and we do not normalize by the underestimated uncertainty. The scatter of the estimated transmission relative to the corresponding linear fit (i.e. the standard deviation of the residuals) across all sub-collimators is 2.5%. This proves that the fitted linear models accurately represent the experimental transmission.

The slope and the intercept (i.e. the on-axis transmission) of the linear fits are contained in Table 1. The linear models plotted in Figure 3 represent our new estimate of the sub-collimator transmission at low energies. Due to a selection bias of the flaring events (see the top-left panel of Figure 2), this transmission model is valid only for offset angles ranging approximately between − 0.5^∘^ and 0.5^∘^.

**Table 1:** Parameters of the linear fits presented in Figures 3 and 5. The slope is reported in units of $10^{-2} \cdot \mathrm{deg}^{-1}$.

	a		b		c	
	On axis transm.	Slope	On axis transm.	Slope	On axis transm.	Slope
10	0.2358 ± 0.0002	−0.06 ± 0.07	0.2418 ± 0.0002	−1.20 ± 0.04	0.2457 ± 0.0002	−0.11 ± 0.08
9	0.2396 ± 0.0002	0.29 ± 0.10	0.2470 ± 0.0002	0.14 ± 0.05	0.2404 ± 0.0002	0.58 ± 0.06
8	0.2164 ± 0.0002	−0.19 ± 0.11	0.2255 ± 0.0002	−0.14 ± 0.05	0.2381 ± 0.0002	−0.29 ± 0.05
7	0.1999 ± 0.0002	−0.13 ± 0.07	0.2105 ± 0.0002	−0.33 ± 0.06	0.2231 ± 0.0002	0.40 ± 0.04
6	0.1996 ± 0.0002	0.73 ± 0.05	0.1854 ± 0.0002	−1.58 ± 0.08	0.1973 ± 0.0002	−0.32 ± 0.04
5	0.1879 ± 0.0001	0.64 ± 0.04	0.2058 ± 0.0002	−2.23 ± 0.11	0.1768 ± 0.0001	−0.09 ± 0.04
4	0.1965 ± 0.0002	−0.24 ± 0.04	0.1967 ± 0.0002	0.65 ± 0.07	0.2105 ± 0.0002	1.42 ± 0.06
3	0.2717 ± 0.0002	2.30 ± 0.05	0.2722 ± 0.0002	0.47 ± 0.06	0.2978 ± 0.0002	2.35 ± 0.12
2	0.1309 ± 0.0001	1.64 ± 0.03	0.0763 ± 0.0001	0.94 ± 0.02	0.1839 ± 0.0002	1.99 ± 0.10
1	0.1165 ± 0.0001	2.45 ± 0.04	0.0384 ± 0.0000	0.93 ± 0.01	0.0908 ± 0.0001	2.43 ± 0.04

The blue lines represent the product of the transmission of the front and rear grids derived from the optical grid measurements performed before the flight. The zero point (offset angle equal to 0) is derived from the optical characterization described in Kuhar (2018) and the decrease reflects the grid shadowing effect, which is computed assuming a perfect grid geometry as follows. Denoted with d the full thickness of the grid, which is nominally equal to 400 μm for the coarse-resolution sub-collimators, the decrease in effective slit width due to internal shadowing is

4 $$ \Delta s = d \cdot \tan \vert \theta \vert \simeq d \cdot \vert \theta \vert ~, $$

where θ is the offset angle defined in Equation 3. Therefore, the effective transmission of a single grid is given by (s−Δs)/p, where s and p denote the slit width and the pitch, respectively.^3^ It follows from Equation 4 that the transmission of a single grid is linear in $\vert \theta \vert $, while the sub-collimator transmission plotted in blue in Figure 3 is quadratic in $\vert \theta \vert $. Hence, the blue lines are in fact two branches of parabola intersecting at θ=0. Given the small range of offset angles displayed Figures 3, the curvature of the parabolas cannot be visually appreciated.

The observed transmission (red points in Figure 3) is systematically lower than that estimated by means of the optical grid characterization by Kuhar (2018) and plotted with blue lines. As we will discuss in detail in Section 3.2, this can be attributed to etching and stacking imperfections.

### Fine-Resolution Sub-Collimators

A similar self-calibration of the transmission has been performed for the six fine resolution sub-collimators, which are labelled as 1a,b,c and 2a,b,c (Krucker et al. 2020; Massa et al. 2023). The grids of these sub-collimators have a nominal pitch of 38 and 54 μm, respectively. Given the difficulty of etching such narrow slits in the available tungsten layers with 33 μm thickness, these grids were fabricated differently from those of the coarsest sub-collimators. Specifically, the grids of sub-collimators 2a,b,c were produced in such a way that their layers contain half of the nominal slats and large slits. The different layers are then stacked so that every missing slat in one layer is present in the adjacent layers (above and below). Overall, the resulting grid has a pitch close to the nominal one but half of effective tungsten thickness. A similar process has been applied for the fabrication of grids 1a,b,c, although three layers are used to create the nominal pitch instead of two. As a result, the effective tungsten thickness is one third of the nominal $d=400~\mu\mathrm{m}$. The different effective thickness of the grids of sub-collimators 1a,b,c and 2a,b,c has an impact at high energies, and appropriate corrections, which will not be discussed in this paper, must be applied. Further, differently from the coarse sub-collimators, the grids of sub-collimators 1a,b,c and 2a,b,c are covered by Kapton layers as a safety measure (see Krucker et al. 2020, for further details), and these layers affect the sub-collimator response at low energies.

To account for the presence of Kapton layers, which make the response of sub-collimators 1a,b,c and 2a,b,c different from that of the CFL in the energy range 10 – 15 keV, we implement the following correction. We consider a typical photon spectrum $\phi (\varepsilon ) \propto \varepsilon ^{-\delta}$, where we set δ=6. Then, we compute the attenuation factor of the Kapton layers between 10 and 15 keV as

5 $$ {\mathrm{Att}}_{\mathrm{K}} = \frac{ \int _{10}^{15} \phi (\varepsilon ) \, t_{K}(\varepsilon ) \,d\varepsilon}{\int _{10}^{15} \phi (\varepsilon ) \,d\varepsilon} \simeq 0.937 ~, $$

where $t_{\mathrm{K}}(\varepsilon )$ is the transmission of the Kapton layers as a function of energy ε. Finally, we compute the empirical transmission of the fine-resolution sub-collimators 1a,b,c and 2a,b,c as

6 $$ t_{\mathrm{fine}} = \frac{F}{F_{\mathrm{CFL}} \cdot {\mathrm{Att}}_{\mathrm{K}}} ~, $$

where F and $F_{\mathrm{CFL}}$ are defined as in 2. Note that, if the power-law index δ varies between 3 and 10, the resulting Kapton attenuation factor (Equation 5) differs from that for δ=6 less than 1%. Therefore, the use of δ=6 is well justified.

**Figure 5:**
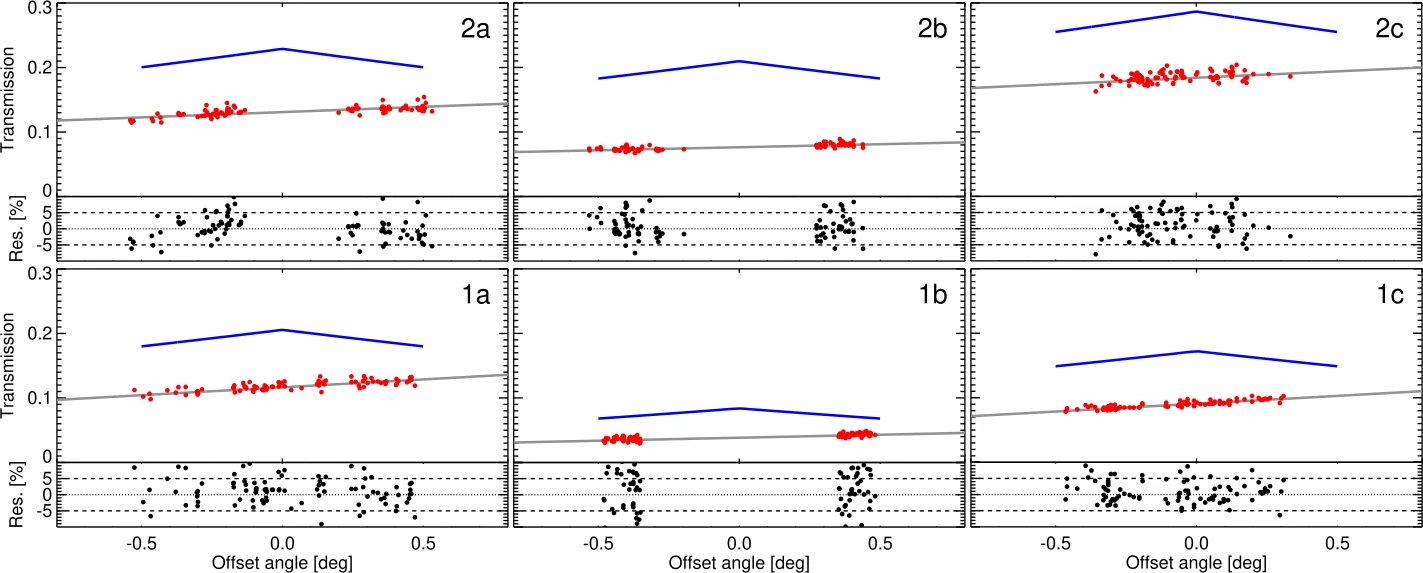
Analogous of Figure 3 in the case of the finest resolution sub-collimators (1a,b,c and 2a,b,c), whose grids are manufactured differently than those of the 24 imaging sub-collimators with coarsest resolution. Note that transmission values are significantly lower than those plotted in Figure 3.

Figure 5 shows the empirical transmission values (red dots) for sub-collimators 1a,b,c and 2a,b,c and the old implementation of the grid transmission based on the on ground grid characterization (blue lines). The linear fits representing the new transmission model we derived are plotted with gray lines, while the residuals (computed in the same way as in Section 2.2) are displayed as black dots. Slope and intercept values of the linear fits are reported in the bottom part of Table 1. Note that the transmission of the fine-resolution sub-collimators is much lower than that of the coarse resolution sub-collimators, especially in the case of sub-collimator 1b. This is due to technical difficulties in manufacturing the finest grids.

Residuals are larger in the case of the six fine-resolution sub-collimators, their standard deviation across all sub-collimators being 4.8%. This indicates that the transmission of these sub-collimators is affected by larger systematic errors compared to those of the coarse-resolution sub-collimators. A possible explanation is that even small imperfections of the grids have a large impact on the transmission as the grid pitch is very small in this case.

### Discussion on the Linear Transmission Model

The new transmission model displayed in Figures 3 and 5 relies on a linear fit to the transmission values computed using flare data. The linear model is certainly a simplification of the actual sub-collimator transmission. However, as shown in Figures 3 and 5, this model is able to capture the trend of the observed transmission neglecting the scatter produced by unknown systematic errors. Further, the residuals displayed in Figures 3 and 5 show that, in most cases, the observed transmission is well represented by a straight line and does not decrease for large absolute values of the offset angle, as it would be expected if the internal shadowing was caused by full grid thickness.

To verify how reliable the simple linear model is for representing the internal shadowing effect, we performed a fit to the observed transmission using a parabola in place of a straight line. A negative concavity of the fitted parabola would be indicative of second-order internal shadowing effects that are not properly accounted for by the linear model. However, the results show that concavity is actually positive for 12 sub-collimators out of 30. Further, for each of the 24 sub-collimators with coarsest resolution (used for spectral analysis), the average relative difference between the linear model and the parabola within the offset angle range defined by the observed flare locations ranges between − 1.1% and + 1.7%. Averaged across this subset of sub-collimators, the relative difference is + 0.1%. In summary, these results demonstrate that internal shadowing is modelled with sufficient accuracy by the simple linear model.

## Effects of Grid Imperfections on the Sub-Collimator Transmission

This Section discusses how stacking and etching imperfections, in combination with a potential tilt of the grid layers, affect the sub-collimator transmission. Specifically, we compare the transmission of a sub-collimator with perfect grids with that of a simulated sub-collimator whose grids are affected by manufacturing imperfections, showing that the on-axis transmission value decreases and that internal shadowing effects are less pronounced. Further, we provide a qualitative explanation for the results displayed in Figure 3.

### Simulation of Stacking and Etching Imperfections

Simple geometric simulations have been performed to illustrate the effect of grid stacking and etching imperfections. From the manufacturing, we know that these imperfections are of the order of a few microns. A single grid is simulated by 8 or 12 individual layers, according to the design of each grid. It is assumed that any part of the grid is fully opaque to X-rays; hence, these simulations are only valid at the low energy end of the STIX energy range. Furthermore, we assume that individual layers have perfectly etched and flat slat walls. This is an unrealistic assumption as the etching process creates substructures. As a result, shadowing effects are enhanced in these simulations compared to reality.

**Figure 6:**
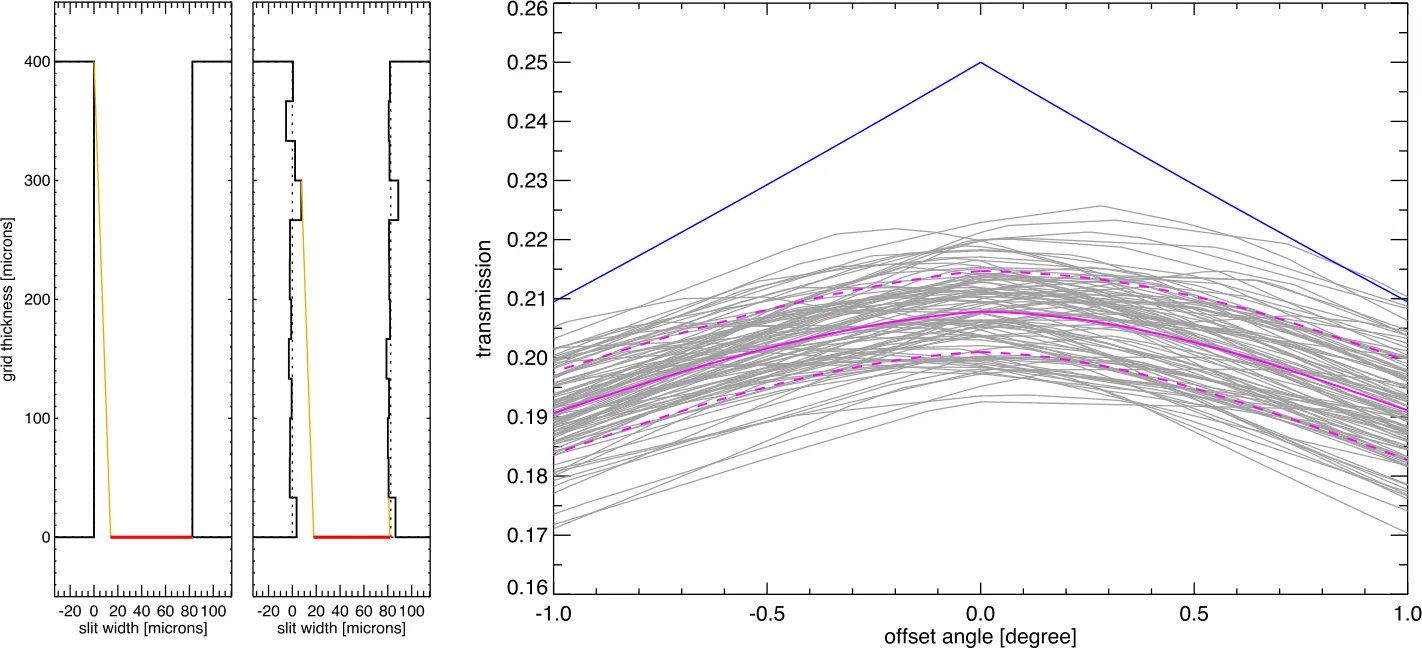
Simulation of the impact of stacking and etching imperfections for low energy photons in the case of grid 5, with a nominal slit width equal to 83 μm and a slit-to-pitch ratio equal to 0.5. *Left panels:* simulation of the effective slit width for a perfect grid (*left*) and for a grid with etching and stacking imperfections (*right*) in the case of X-ray photons with an incident angle of 2^∘^ (the panels show the correct aspect ratio of the grid). The two orange lines indicate extreme cases of incoming X-rays which pass just through the corner of the innermost layer. The red area marks the resulting effective slit width. *Right panel:* transmission through two grids (i.e. a sub-collimator) for 100 random representations of stacking and etching imperfections with a standard deviation of 2 μm. The transmission as a function of the offset angle for the different representations of manufacturing imperfections is plotted as *gray curves*. Note that the curvature of the gray lines is due to the perfectly sharp edges of the simulated grid layers (see text). The *solid magenta line* indicates the average transmission over all simulations, while the *dashed magenta curves* represent their standard deviation. The *blue lines* represent the transmission of a perfect grid.

To simulate the effect on grid transmission, we create 100 different representations of the grid geometry with random imperfections (both etching and stacking) drawn from a Gaussian distribution with standard deviation equal to 2 μm. Figure 6 shows an ideal grid as well as a grid with random stacking and etching imperfections for illustration. The transmission as a function of the offset angle is then calculated using the corners of each layer (see the orange lines in the left and middle panels of Figure 6).

The transmission values associated with the 100 random grid representations are shown in the right panel of Figure 6. The perfect grid is assumed to have an on-axis transmission equal to 0.25 as the slit-to-pitch ratio of both front and rear grid is set equal to 0.5 in the simulation software. The off-axis transmission falls off due to internal shadowing of the grid slats. For the grids with imperfections, the transmission changes: the on-axis transmission is reduced as some layers extend into the slit area and the internal shadowing effect is less prominent (or essentially absent) in the case of small incident angles of the photons. In particular, the curved transmission profiles are due to the simulated sharp edges of the layers, which are not reflecting the results by an actual etching process. The solid magenta line gives an average transmission, but such an average can deviate largely from an individual realization of imperfections and therefore we cannot use it to derive an empirical correction. Figure 6 shows that simulated stacking and etching imperfections with a standard deviation of 2 μm lead to an average reduction of the transmission to 83% for the case of sub-collimator 5 with a slit of 83 μm and a pitch of 166 μm. For a single grid, this translates to a slit width reduction of ∼ 6 μm to 76 μm for the on-axis case.

In addition to random stacking imperfections there could be systematic stacking imperfections as well, which can result in a tilted slit or, in 3D, in a spiral staircase profile. In our 2D simulation, a tilt results in a shift of the transmission maximum, with the peak transmission at the tilt angle (similarly to what shown in the right panel of Figure 19 in Su et al. 2024). A tilt of half a degree for a 400 μm thick grid requires a shift from top to bottom layer of ∼ 3.5 μm. Such a value is of the same order as the maximal stacking imperfection, and therefore plausible. For low energy radiation, an opposite shift of the top and bottom layers can result in the same transmission as a tilted grid where all layers show the tilt. For cases with a large enough tilt, the actual transmission as a function of offset angle can therefore steadily increase or decrease.

### Qualitative Interpretation of the Effective Transmission

**Figure 7:**
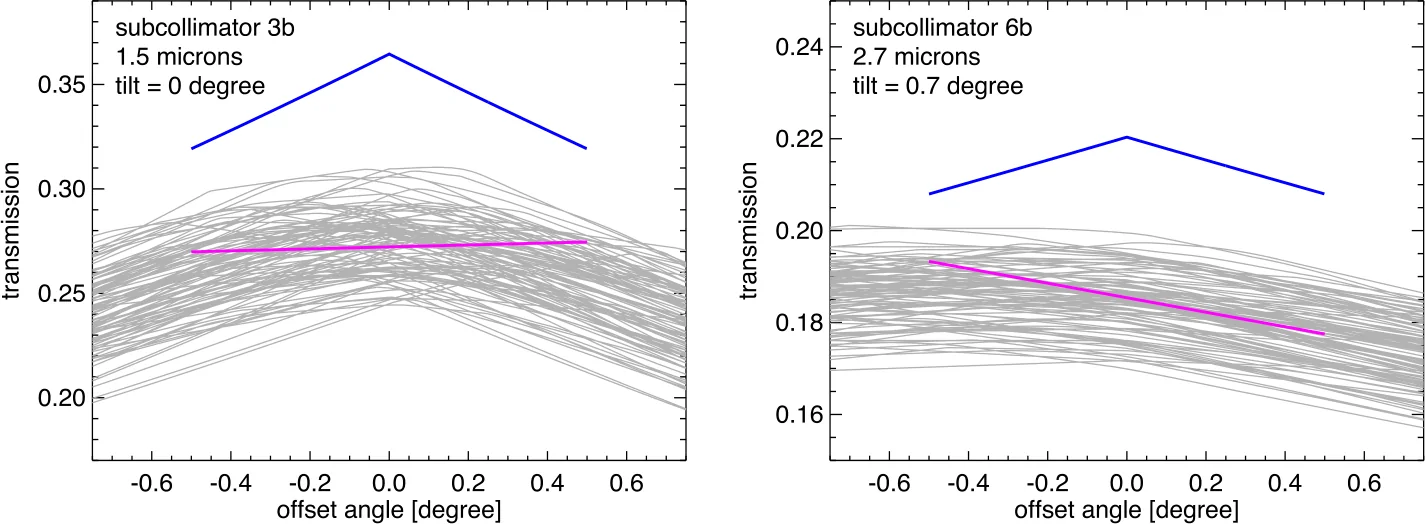
Comparison between the derived sub-collimator transmission as a function of the offset angle and simulations. Two extreme cases are shown: sub-collimator 3b with a large reduction in transmission (*left panel*) and sub-collimator 6b with a large slope (*right panel*). The values of simulated etching and stacking imperfections, as well as that of the grid tilt, are reported in the legend. The fitted linear models and the grid transmission profiles derived from the on ground grid characterization (see Figure 3) are shown in *magenta* and *blue*, respectively. The *gray* curves represent 100 random simulations for the parameter values reported in the top-left corner.
- Sub-collimator 6b shows a clear slope in the transmission as a function of the off-axis angle (see Figure 3). A simulation with a tilt of the order of 0.7 degree in combination with stacking and etching imperfections of 2.7 μm gives a similar trend, although the observed slope is slightly steeper. A 0.7-degree tilt produces a shift of the peak transmission equivalent to that caused by a 5 μm shift of the top and bottom layers. In units of the pitch, it is a 2% shift. This is a significant deviation from the desired value. Such imperfections can occur when the alignment pins used in the stacking process are tilted from the normal direction.

We use the simple simulations discussed in Section 3.1 to get a qualitative description of the newly established self-calibrated transmissions. The discussion is not meant to give a quantitative explanation, but rather a plausibility argument. Figure 7 shows two extreme examples that we discuss in the following.

- Sub-collimator 3b shows a large reduction in grid transmission of about a fourth and the grid shadowing effect is rather small in the new calibration, despite that the slit to thickness ratio is rather small (∼ 0.1). The reduction in transmission can be attributed to stacking and etching imperfections, each of the order of 1.5 μm. Considering the fabrication methods of the grids, such imperfections are plausible and could not be seen with the optical calibration done at 2 μm pixel size. Stacking imperfections are the main contributors to the reduction in grid transmission, assuming that the amount of stacking and etching imperfections is comparable. Stacking imperfections alone with a standard deviation of 1.5 μm give already a reduction similar to that shown in the left panel of Figure 7. For etching imperfections without any stacking imperfections, the etching imperfection would need to be more than double to achieve a similar reduction in transmission.

In summary, stacking and etching imperfections in combination with a tilt give a plausible explanation for the difference between the optical grid calibration and the value obtained by self-calibration. However, this explanation is not conclusive and the presence of other effects cannot be excluded.

## Consistency Tests

Below we present the results of two different consistency tests we performed using STIX solar flare observations. The outcomes of these tests allow us to quantify the degree of reliability of the new transmission calibration and to determine the differences with respect to the old calibration.

### Consistency Test on an Independent Set of Events

To quantify the reliability and robustness of the new transmission calibration when applied to a new set of events, we select 25 flaring events that occurred between March and December 2025 and are not included in the dataset utilized for the transmission calibration. For each flare, we consider an appropriate integration time in such a way that the total number of counts recorded by the 24 coarse-resolution sub-collimators is larger than $2 \cdot 10^{5}$. We apply the ELUT correction to the counts recorded between 10 and 15 keV, we divide the counts in each time bin by the corresponding live time, and we compute the average of the resulting count rates. We sum the count rates recorded by the eight large pixels of each sub-collimator detector, and divide the result by the sub-collimator transmission. We apply two versions of the sub-collimator transmission: the new transmission derived from the linear fits shown in Figure 3 and the old transmission based on the on-ground grid characterization (blue lines in Figure 3). Finally, for each flaring event, we normalize the total flux recorded by each individual sub-collimator by the average across the different detectors.

**Figure 8:**
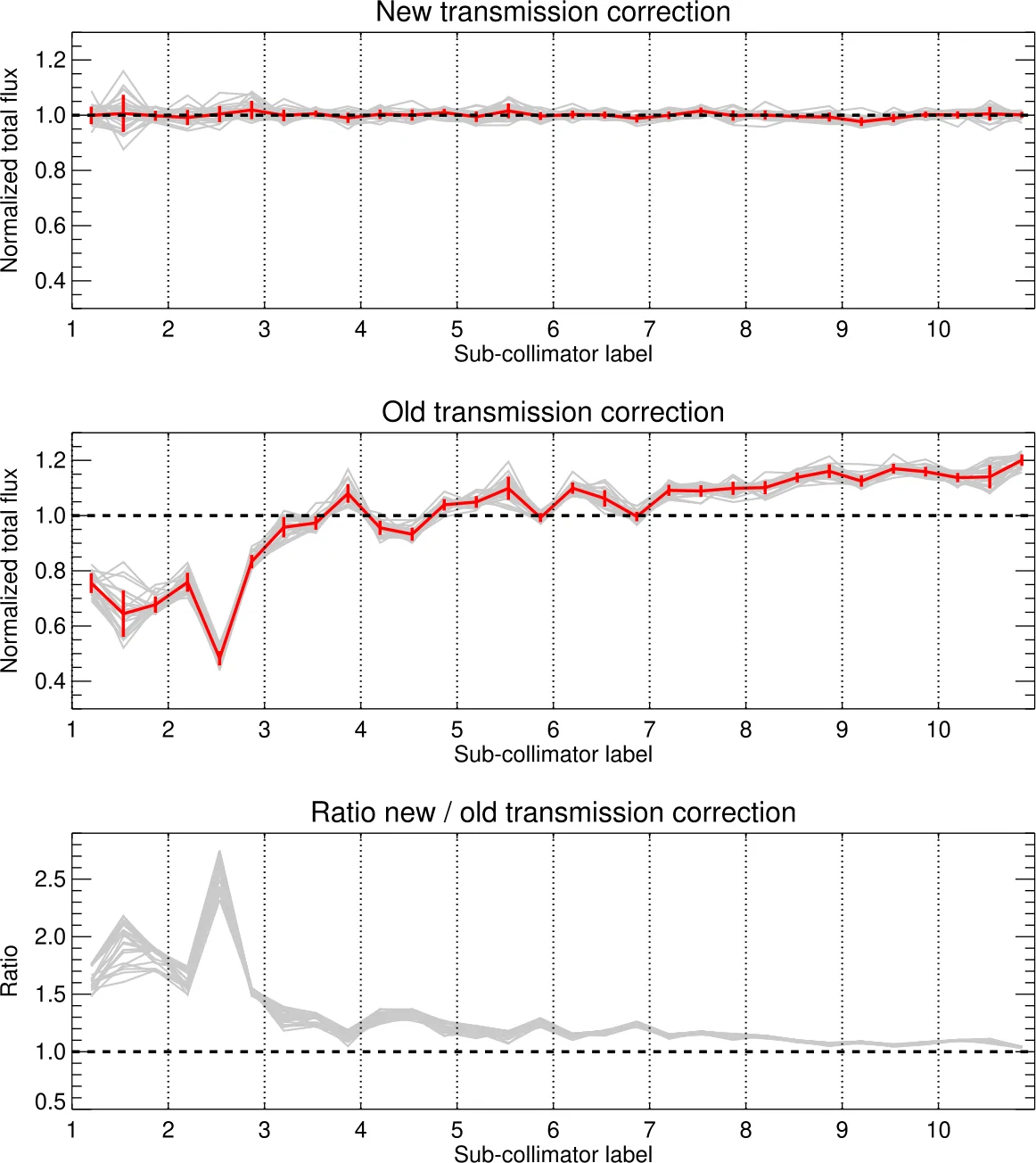
*Top panel:* total flux recorded by the imaging sub-collimators (i.e., A+B+C+D) corrected for the transmission derived from the linear fits of Figure 3, and normalized by the average across the different sub-collimators. Each of the 25 gray lines corresponds to a flaring event recorded between March and December 2025. The sub-collimators with the same label number are ordered as a, b, and c, from left to right, respectively. For each sub-collimator, the average of the normalized total flux over the considered set of events is plotted in red, and the standard deviation is overlaid as a vertical bar. As a reference, we plot a black dashed line at height equal to 1, which indicates a perfect agreement between the total flux estimates provided by the different sub-collimators. *Middle panel:* same plot as in the *top panel* but for the old transmission values (blue lines in Figure 3). *Bottom panel:* ratio between the total flux corrected for the new grids’ transmission (derived from the linear fits shown in Figure 3) and the old grids’ transmission for the same events as shown in the top panels. A dashed constant line equal to 1, representing a perfect match between the old and the new version of the grids’ transmission, is plotted in black as a reference.

The top panel of Figure 8 shows the normalized total flux values obtained with the new transmission calibration for each of the 25 flaring events, represented by the gray lines. For each sub-collimator, the average of the normalized total flux values across the different events is plotted in red, and the standard deviation is overlaid as a red error bar. The average normalized total flux values range between 0.98 and 1.02, showing consistency among the different sub-collimators. Further, despite being based on a simple linear model, the new transmission calibration is robust when applied to different events within the full-sensitivity STIX FOV. The standard deviation of the normalized total flux values across the different events is below 3.3% for all the detectors except for 1b, whose standard deviation is equal to 6.7%.

As for the normalized total flux values obtained with the old transmission calibration (gray lines in the middle panel of Figure 8), each individual sub-collimator shows a scatter comparable to (although slightly larger than) that obtained with the new transmission calibration, their standard deviation (red error bars) being below 4.2% for all sub-collimators except for 1b that shows a standard deviation of 8.4%. However, these normalized total flux values show a large systematic bias: their average values over the different events in the dataset (red line) range between 0.93 and 1.20 for the 24 coarsest sub-collimators labelled from 3 to 10, and between 0.48 and 0.86 for the six finest sub-collimators.

The bottom panel of Figure 8 displays the ratio between the total flux values obtained with the old and the new transmission calibration for each flaring event. The results show that the fluxes normalized by the new transmission are consistently larger than those normalized by the old transmission, their relative difference ranging between 3% and 39% for the 24 coarsest-resolution sub-collimators and between 48% and 175% for the six finest-resolution sub-collimators. The new sub-collimator transmission leads to a dramatic improvement for the finest-resolution sub-collimators. In the case where only half of the sub-collimators with coarsest resolution (labelled from 10 to 7) would be considered, which are less sensitive to effects of grid imperfections on the transmission (see Figure 3), the average relative difference between the total flux values obtained with the new and the old transmission would be ∼ 10%.

### Consistency Test Across Different Sub-Collimators

We evaluate the consistency among spectral fit results independently obtained from the different sub-collimators with the following test. We select the X2.2 GOES class flaring event SOL2024-12-08T09:05, which is outside of the set of events considered for the calibration task (see Section 2.1) and it presents excellent counting statistics. The coordinates of the flare location, expressed with respect to the HPC reference frame, are $x=924^{\prime \prime }$ and $y=-227^{\prime \prime }$. The same location, expressed with respect to the STIX imaging frame, which is the relevant frame for the computation of the internal shadowing, corresponds to $x=-148^{\prime \prime }$ and $y=-922^{\prime \prime }$. We consider the time range between 09:06:24 and 09:07:06 UT (at Solar Orbiter) which is during the early decay phase when thermal emission dominates. We select the energy range between 10 and 15 keV, and we fit a single isothermal model to each spectrum provided by an individual sub-collimator. We then use the fitted models to convert the recorded count spectra to the corresponding photon spectra. The spectral fit is performed by means of the Object Spectral Executive (OSPEX; Tolbert and Schwartz 2020).

**Figure 9:**
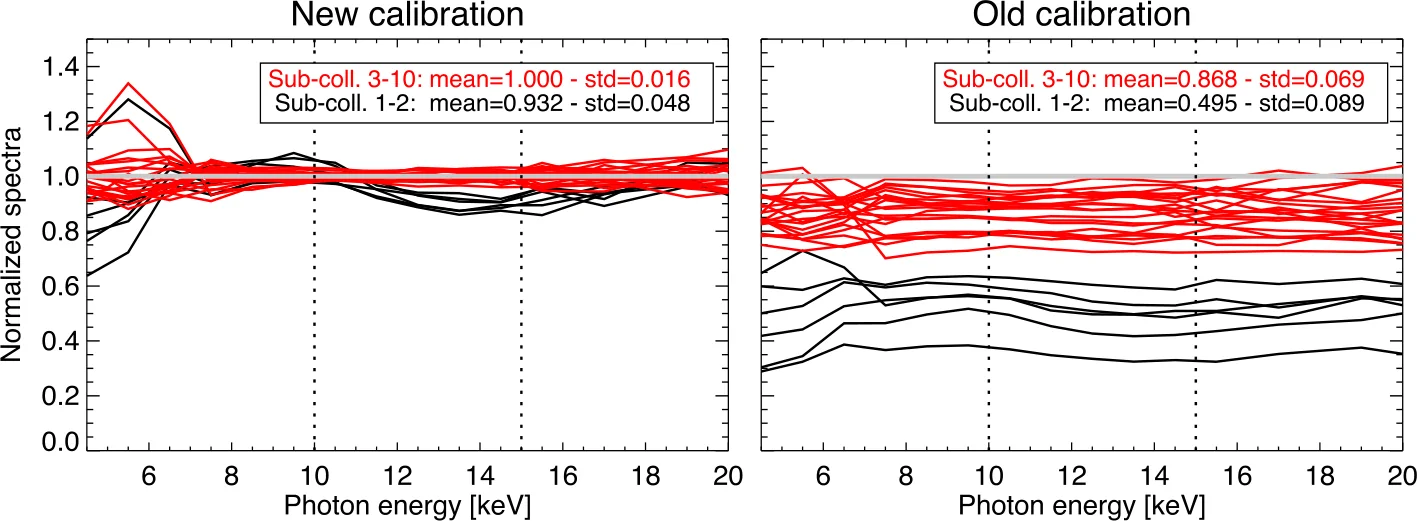
Comparison between the photon fluxes derived using the old and the new calibration for the GOES X2 flare SOL2024-12-08. *Left panel:* normalized photon spectra obtained by independently fitting the count spectra provided by each individual sub-collimator (excluding subcollimators 3a, 5a, and 5b, see text for details) using the new transmission calibration. The *red lines* indicate the photon spectra corresponding to the 21 coarse-resolution sub-collimators, while the *black lines* indicate photon spectra derived from the six fine-resolution sub-collimators. Each line is normalized by the average of the photon spectra of the 21 coarse-resolution sub-collimators. The average and the standard deviation of the normalized spectra between 10 and 15 keV are reported in the legend. *Right panel:* same as in the left panel in the case of the old transmission calibration. For a meaningful comparison the spectra are again normalized by the average of the photon spectra of the 21 coarse-resolution sub-collimators obtained with the new calibration.

Figure 9 shows a comparison of the photon spectra derived from each individual sub-collimator using the new and the old transmission calibration (left and right panel, respectively). The spectra are normalized by the average value of those fitted from the coarse-resolution sub-collimators (with label from 3 to 10) using the new transmission calibration, which are plotted in red in the left panel. While performing this test, we noticed that the spectra obtained from sub-collimators 3a, 5a, and 5b show an energy-dependent discrepancy with respect to those provided by the remaining 21 coarse-resolution sub-collimators. Therefore, sub-collimators 3a, 5a, and 5b have been excluded from the consistency test shown in Figure 9. This discrepancy is likely not related to the grid calibration, but rather to the detector calibration, and will be investigated in a separate work. For now we recommend excluding these sub-collimators when performing spectral analyses.

The results presented in Figure 9 show that the spectra obtained with the old calibration from the coarse-resolution sub-collimators are on average 13.2% lower than those obtained with the new calibration. This is a direct consequence of the fact that the old calibration consistently overestimates the sub-collimator throughput,^4^ as highlighted in Figure 3, and has an impact on the estimation of the emission measure (EM). With the new calibration, the fitted EM is indeed 13.7% larger than that obtained with the old transmission calibration. Further, consistency among the photon spectra corresponding to sub-collimators 3 to 10 increases when the new sub-collimator transmission is considered. In fact, the standard deviation of the spectra values in the 10 – 15 keV range decreases from 6.9% (old calibration) to 1.6% (new calibration).

Similar considerations apply for the six fine resolution sub-collimators (labelled as 1 and 2), which are plotted in black in Figure 9. The improvement resulting from the new sub-collimator transmission is much larger compared to that of the coarse-resolution sub-collimators, as the average of the normalized spectra values increases from 0.51 (old calibration) to 0.96 (new calibration). However, the photon spectra obtained with the new calibration show an evident energy-dependent bias. A correction will be investigated in a future study concerning the behaviour of the grid transmission as a function of photon energy. For now we recommend excluding the six fine-resolution sub-collimators when performing spectral analyses.

It can be appreciated from the results presented in the left panel of Figure 9 that, although the grid transmission calibration has been performed between 10 and 15 keV, the spectra derived from the individual sub-collimators are consistent in a larger energy interval from ∼ 8 to ∼ 20 keV. The standard deviation of the spectra values obtained from the coarse-resolution sub-collimators with the new transmission calibration is 2.3% in the extended energy range.

The spectra plotted in Figure 9 present an increased scatter below 8 keV. This effect is possibly due to inhomogeneities in the Solar Black coating, which could affect the transmission at low energies in a different way for the different sub-collimators. If this is indeed the case, these deviations should show a dependency on the flare location, as the amount of transmitted radiation falling onto the detector should depend on the incident angle. Hence, there is a way to potentially self-calibrate the Solar Black transmission by using a large number of observed flares. Such a study is outside the scope of this paper, but well worth looking into.

## Summary and Conclusions

In this work we presented a self-calibration procedure from in-flight data to accurately determine the STIX sub-collimators’ transmission at low energies ($\lesssim 20\text{ keV}$) for offset angles ranging between − 0.5^∘^ and 0.5^∘^. The calibration task is performed by considering a dataset of events for which at least one of the CFL detector pixels is fully illuminated by the flaring X-ray radiation and can be used as a total flux monitor.

Our results show that the effective transmission of the imaging sub-collimators is lower than what was predicted from a model relying on grid parameter values that were characterized on the ground via optical measurements. Further, internal shadowing effects are much less pronounced than what was expected from a model assuming a perfect grid. Simulations of stacking and etching imperfections of the grid layers show that the reduced transmission and internal shadowing impact can be explained by imperfections of the order of a few microns on each side of the slats. Such imperfections are plausible given the accuracy of the manufacturing process.

Results obtained considering flaring events outside of the calibration dataset show that the new transmission calibration improves the consistency between the total flux estimates and the spectra provided by the different sub-collimators. The standard deviation of the photon spectra values fitted from each individual sub-collimator is ∼ 2% between 8 and 20 keV. While the relative calibration of individual STIX sub-collimators is now well established, absolute calibration should be performed in the future by comparing measurements of the same flares by Earth-orbiting observatories when Solar Orbiter is near the Sun-Earth line.

The new calibration leads to reconstruction of photon spectra that are ∼ 13% larger than the corresponding ones obtained with the old calibration, and the EM values fitted with the new calibration are larger by a similar amount. Nonetheless, conclusions derived in previous papers from STIX spectral analyses performed with the old calibration are not affected. Imaging results are even less affected as corrections on the grid transmission calibration have a second order effect on the calibration of the visibility amplitudes. As an example, for a sub-collimator with grids with slit-to-pitch ratio equal to 0.5, 10% corrections on the grid transmission calibration would result into a 2.5% correction on the corresponding visibility amplitude (see Equations (9), (16), and (18) in Massa et al. 2023). Hence, only the amplitude calibration of the fine-resolution sub-collimators is significantly affected as, for these sub-collimators, uncertainty on the transmission is significantly larger than 10% (see the bottom panel of Figure 8). The visibility amplitude calibration based on the results presented in this paper will be released in the upcoming version of the STIX analysis software.

The transmission calibration described in this paper has been performed only at low energies. However, it represents a necessary step for the high-energy grid calibration, which will be addressed in a future work. The high-energy calibration is crucial for the derivation of conclusive results regarding the open issue of the anisotropy of the non-thermal emission, which needs to be addressed by comparing spectral results from instruments with different viewing angles on the flaring site. It has been shown that an instrument cross-calibration more accurate than 10% is needed to derive meaningful results (Jeffrey et al. 2024), and this study represents a necessary step towards this goal.

As a final remark, we strongly recommend future indirect X-ray imagers to include a dedicated total flux monitor, in such a way that every recorded flare can be used for self-calibration purposes. Furthermore, a total flux monitor with sufficiently large effective area would allow performing the same calibration procedure presented in this paper also at high energies. Total flux monitors are included in HXI and are currently adopted for calibration tasks (e.g., Su et al. 2024).

## Data Availability

The STIX data analyzed in the current study are available at the Solar Orbiter Archive (https://soar.esac.esa.int/soar).

## Footnotes

1 https://github.com/hayesla/stix_flarelist_science.git.

2 The integer number of counts registered by the STIX pixels are compressed onboard to an 8 bit representation to save telemetry. These numbers are decompressed on the ground and the uncertainty associated with the compression scheme is propagated together with the statistical uncertainty. For the sake of simplicity, compression uncertainty is not described in the main text, although it is properly taken into account in the data analysis we performed.

3 Sub-collimators with labels from 1 through 8 contain *bridges*, which are stripes of tungsten displaced orthogonally to the grid slats (see Figure 3 in Massa et al. 2023). The width and separation of the bridges were optically measured by Kuhar (2018), and this characterization is utilized to account for the presence of bridges in the sub-collimator transmission.

4 A count spectrum φ(q) and the corresponding photon spectrum $\phi (\varepsilon )$ are related by

$$ \varphi (q) = \int \phi (\varepsilon ) \cdot {\mathrm{SRM}}(q,\varepsilon ) \,d\varepsilon ~, $$

where the Spectral Response Matrix (${\mathrm{SRM}}$) includes information on the grid transmission. Therefore, the fitted photon spectra are inversely proportional to the grid transmission value.
